# H1N1 influenza vaccination in HIV-infected women on effective antiretroviral treatment did not induce measurable antigen-driven proliferation of the HIV-1 proviral reservoir

**DOI:** 10.1186/s12981-017-0135-1

**Published:** 2017-02-13

**Authors:** Thor A. Wagner, Hannah C. Huang, Christen E. Salyer, Kelly M. Richardson, Adriana Weinberg, Sharon Nachman, Lisa M. Frenkel

**Affiliations:** 1 University of Washington, Seattle, USA; 2 Seattle Children’s Research Institute, 1900 Ninth Ave, 8th Floor, Seattle, WA 98101 USA; 3 Indiana University, Indianapolis, USA; 4 University of Colorado Anschutz Medical Center, Aurora, USA; 5 State University of New York at Stony Brook, Stony Brook, USA

**Keywords:** HIV, HIV DNA, Influenza, Vaccination, H1N1, Pregnancy, Antiretroviral therapy, Latency, Antiretroviral treatment

## Abstract

**Objectives:**

Antigen-induced activation and proliferation of HIV-1-infected cells is hypothesized to be a mechanism of HIV persistence during antiretroviral therapy. The objective of this study was to determine if proliferation of H1N1-specific HIV-infected cells could be detected following H1N1 vaccination.

**Methods:**

This study utilized cryopreserved PBMC from a previously conducted trial of H1N1 vaccination in HIV-infected pregnant women. HIV-1 DNA concentrations and 437 HIV-1 C2V5 *env* DNA sequences were analyzed from ten pregnant women on effective antiretroviral therapy, before and 21 days after H1N1 influenza vaccination.

**Results:**

HIV-1 DNA concentration did not change after vaccination (median pre- vs. post-vaccination: 95.77 vs. 41.28 copies/million PBMC, p = .37). Analyses of sequences did not detect evidence of HIV replication or proliferation of infected cells.

**Conclusions:**

Antigenic stimulation during effective ART did not have a detectable effect on the genetic makeup of the HIV-1 DNA reservoir. Longitudinal comparison of the amount and integration sites of HIV-1 in antigen-specific cells to chronic infections (such as herpesviruses) may be needed to definitively evaluate whether antigenic stimulation induces proliferation of HIV-1 infected cells.

**Electronic supplementary material:**

The online version of this article (doi:10.1186/s12981-017-0135-1) contains supplementary material, which is available to authorized users.

## Background

A cure for human immunodeficiency virus type-1 (HIV-1) infection may hinge on understanding the mechanisms that allow HIV-1 to persist during otherwise effective antiretroviral treatment (ART). Studies of HIV-1 C2-V5 *env* and *pol* DNA and RNA sequences and integration sites during effective ART have shown multiple identical *env* and *pol* templates (which we describe as “monotypic”) [1, 2], an increase in the proportion of monotypic *env* and *pol* proviruses over time [2, 3], and a linkage between monotypic *env* through the 3′ LTR proviral sequences with identical chromosomal integration sites [4], all indicating that HIV-infected cells proliferate. This study addressed our hypotheses that proliferation of HIV-1 infected CD4+ T-cells occurs at least in part by exposure to recall antigens, and that antigen-driven proliferation contributes to sustaining the HIV-1 reservoir. We took advantage of a H1N1 influenza vaccination study in HIV-1-infected pregnant women to explore this hypothesis.

Immunological responses to infections [5] and vaccines [6–8] have been associated with increases in HIV-1 plasma viral load, although the effect of influenza vaccination on plasma HIV-1 RNA has been variable [9–12]. The effect of vaccination on the proviral population has not been explored in detail. Studying responses to H1N1 influenza vaccination in pregnant HIV-infected, ART-treated, women who were vaccinated against H1N1 in last few months of 2009 provided a unique opportunity to explore the effect of recall antigen response on the HIV DNA population. The H1N1 pandemic strain emerged in the first few months 2009, and showed antigenic similarities to a strain that circulated in the human population in the mid 1970s [13]. As this strain presented neo-antigens to those born after the mid-1970s, including the study population of HIV-infected women, the study design presumed that participants with a pre-vaccination pandemic H1N1-specific antibody response had been infected and developed an immune response during the 2009 pandemic. However, because the pandemic H1N1 had approximately 70% homology at the T cell epitope level with recent seasonal H1N1 serotypes, very low pre-vaccination responses were considered due to cross-reactivity. Participants who were not on ART in the first half of 2009 during the H1N1 pandemic and had serologic evidence of infection with H1N1 antigens, were expected to have a recall response to the vaccination. Women who subsequently started ART should have proliferation of influenza-specific CD4+ memory T cells after vaccination, some of which would be latently infected with HIV-1. Increases in proviruses due to T-cell proliferation would cause an increase in the proportion of monotypic sequences in the viral population. In contrast, women on effective ART when they were infected with pandemic H1N1 or without pre-existing H1N1 antibody titers would not be expected to have evidence of proliferation of HIV-infected cells after H1N1 vaccination.

To explore whether a response to a recall antigen leads to proliferation of HIV-1 infected cells, the HIV DNA from PBMC was quantified, multiple HIV-1 DNA genetic sequences were generated from each participant from before and after vaccination, and these sequences were analyzed for evidence of proliferation of HIV-infected cells. Because the timing of HIV diagnosis, ART, primary H1N1 antigen exposure, and subsequent H1N1 vaccination are well defined for this cohort, we had the potential opportunity to investigate the specific effect of antigen-driven cell proliferation on HIV persistence during ART.

## Methods

This study utilized demographic and antibody data and specimens from HIV-1 infected pregnant women who had participated in a Phase II Study to Assess the Safety and Immunogenicity of an Inactivated Swine-Origin H1N1 Influenza Vaccine in HIV-1 Infected Pregnant Women (IMPAACT P1086) [14]. As part of P1086, participants’ blood was collected before and 21 days after the first H1N1 vaccination (high dose, 30 μg), at which time (21 days post primary vaccination) they received a H1N1 booster. Participants were selected for this substudy based on: (1) ART-suppression of HIV-1 replication (plasma HIV-1 RNA <50 copies/mL, or plasma HIV-1 RNA <500 copies/mL and decreasing in those who started ART approximately at the same time as enrolling into the study), and (2) specimens available for analysis from before and after vaccination. P1086 study participants were not selected based on pre-existing antibody response to H1N1. H1N1 had entered the US beginning ~9 months prior to the P1086 study, therefore P1086 study enrolled a mixture of participants with and without immunity to H1N1 prior to H1N1 vaccination. Utilizing specimens from this H1N1 vaccination trial allowed a unique opportunity to investigate the HIV population immediately before and after a defined antigen stimulus in a mixed population for which H1N1 was a recall antigen for some individuals and a de novo antigen for others. Study endpoints included (1) PBMC DNA load by limited dilution PCR, (2) proportion of monotypic sequences defined as ≥2 identical HIV C2-V5 *env* sequences, and (3) quantification of low-level plasma HIV RNA defined as ≥9 c/mL.

DNA was extracted from peripheral blood mononuclear cell (PBMC) samples using a kit (5 Prime ArchivePure DNA Kit; 5 PRIME Inc., Gaithersburg, MD) and quantified using spectrometry (NanoDrop 1000 Spectrophotometer; Thermo Scientific, Waltham, Ma). To quantify HIV-1 DNA, the DNA was serially diluted, triplicates of each dilution underwent PCR to amplify HIV-1 *env* C_2_-V_5_, and the Quality program [15] was used to estimate the number of copies of HIV-1 in the sample, using 1st-round (BH2 [16] and Env6834 (CAG GCC TGT CCA AAA GTA TCC TTT GAG CCA ATT CC), and 2nd-round (DR7 [17] and DR8 [18]) PCR primers, and cycling conditions previously published [19]. To amplify ~20 single viral templates, the DNA from each specimen was diluted to a concentration expected to yield an amplicon from ~30% of PCR; at this concentration 70% of the amplicons are predicted to be from a single viral template. Sequences that appeared in chromatograms to have been derived from more that one template were discarded.

The amplicons were purified (ExoSAP-IT; Affymetrix, Santa Clara, CA) and 2uL of the cleaned product was added to 2uL of primers DR7 and DR8 at concentration of 3.2 pmol for dideoxynucleotide sequencing. Sequences were analyzed using Sequencher program v5.0.1 (Gene Codes Corporation, Ann Arbor, MI) and aligned in Seaview (http://pbil.univlyon1.fr/software/seaview3.html). DIVEIN was used to construct phylogenetic trees and compute divergence of sequences from the most recent common ancestor (MRCA) and sequence diversity [20]. The trees were rooted against reference subtype B sequences except participant 2 who was infected with a complex recombinant and was rooted against a collection of similar sequences in the HIV Sequence Compendium. FigTree (http://tree.bio.ed.ac.uk/software/figtree) was used to graphically represent the phylogenetic trees.

To determine if H1N1 vaccine was associated with low-level viremias, HIV-1 RNA was quantified from subjects’ plasma using previously published methods [21] with the following adaptations. Viral particles from 4.7 mL of plasma (in Beckman OptiSeal Tubes) were pelleted by ultracentrifugation at 175,000 g for 30 min. Afterwards, 3.7 mL of plasma was carefully removed from above the pellet, leaving the 1 mL of plasma overlying the pelleted virions undisturbed. The pellet was resuspended and the HIV RNA was quantified (Abbott RealTime HIV-1 Assay; Abbott Molecular, Abbott Park, Illinois). The calculated lower limit of quantification after concentration of the virions was approximately 9 copies/mL of plasma.

Comparisons of population medians and proportions were performed using Students t-Test for two samples with 2-sided tails (Excel, Microsoft, Redmond, WA).

## Results

Specimens from 26 participants were processed, but only 10 participants yielded sufficient HIV-1 DNA from before and after vaccination to derive multiple sequences of *env.* Pre- and post-vaccine data were compared across all 10 participants, and between participants separated into two groups based on whether or not each woman was predicted to exhibit H1N1-specific proliferation of HIV-infected CD4+ T-cells. Group 1 included participants predicted to not have proliferation of HIV-infected cells after vaccination; including those with a negative H1N1 titer pre-vaccination, those without evidence of response to vaccination as measured by Hemagglutination Inhibition assay (HAI), and those on suppressive ART since prior to April 2009, the beginning of the H1N1 pandemic. Group 2 included participants that were predicted to have proliferation of HIV-infected cells based on a pre-vaccine H1N1 Influenza HAI titer ≥1:20, a >three fold increase in post vaccine HAI titer, and initiation of ART in or after April 2009. The Group 2 women would have developed a primary response to the H1N1 pandemic strain before ART initiation. These women were HIV-infected at the time of their H1N1 Influenza infection, and would likely have integrated HIV proviruses in H1N1-specific CD4+ T cells. Based on these criteria, three participants were classified as having potential to show antigen-driven proliferation of HIV-infected H1N1-specific cells in response to vaccination, and seven were not expected to demonstrate proliferation of HIV-infected cells following vaccination. Clinical parameters, including CD4+ cell count, plasma HIV-1 RNA at enrollment, and time on ART are summarized in Table 1.

**Table 1:** Summary of clinical parameters and analysis of HIV before after H1N1 vaccination

Subject ID	Time-point	Pandemic H1N1 HAI Titer	Seasonal H1N1 HAI Titer	CD4+ (cells/mm^3^)	Days on ART	Copies HIV DNA/million PBMC	Plasma RNA (copies/mL)	# HIV DNA Seq.	Divergence median (95% CI)	Diversity median (95% CI)	# Mono-typic sequences	% Mono-typic
1	Entry	5	5	140	125	358	112	20	.080 (.047 to 113)	.052 (.007 to .097)	2	10
	Week 3	20	5	–		206	38.4	36	.08 (.060 to .100)	.056 (.019 to .093)	10	28
2	Entry	10	10	337	77	22	ND	18	.023 (.020 to .027)	.032 (.019 to .045)	3	17
	Week 3	20	10	–		17	ND	23	.017 (.014 to .023)	.026 (.014 to .038)	7	30
3	Entry	10	10	475	386 ^a^	398	NA	21	.029 (.018 to .040)	.029 (.012 to .046)	2	10
	Week 3	10	5	–		614	NA	19	.029 (.010 to .048)	.034 (.012 to .056)	4	21
4	Entry	5	10	623	882 ^a^	123	NA	21	.019 (.009 to .029)	.020 (−.001 to .041)	10	48
	Week 3	80	10	–		157	NA	20	.020 (.009 to .031)	.019 (.006 to .032)	6	30
5	Entry	5	40	1383	57	3	NA	22	.024 (.022 to .026)	.019 (.009 to .029)	10	45
	Week 3	160	20	–		10	NA	20	.024 (.017 to .031)	.019 (.010 to .028)	8	40
6	Entry	40	40	400	302 ^a^	24	<8	28	.017 (.006 to .028)	.017 ( to .003.037)	7	25
	Week 3	320	40	–		6	ND	24	.017 (.007 to .027)	.02 (.003 to .037)	8	33
7	Entry	80	40	608	2494 ^a^	70	ND	19	.016 (.010 to .022)	.015 (.004 to .026)	7	37
	Week 3	160	40	–		3	ND	20	.016 (.013 to .019)	.015 (.007 to .023)	6	30
*8*	*Entry*	*20*	20	*381*	*106*	*52*	*11.4*	24	*.054 (.023 to .085)*	*.037 (.004 to .070)*	*8*	*33*
	*Week 3*	*160*	*20*	–		*57*	*<8*	*23*	*.052 (.034 to .070)*	*.042 (.010 to .074)*	*6*	*26*
*9*	*Entry*	*20*	*40*	*501*	*56*	*122*	*ND*	*21*	*.045 (.013 to .077)*	*.045 (.020 to .070)*	*8*	*40*
	*Week 3*	*160*	*20*	–		*186*	*ND*	*20*	*.049 (.027 to .071)*	*.043 (.019 to .067)*	*8*	*40*
*10*	*Entry*	*80*	*20*	*712*	*85*	*153*	*NA*	*20*	*.050 (.016 to .084)*	*.059 (.022 to .096)*	*5*	*25*
	*Week 3*	*1280*	*20*	–		*26*	*NA*	*24*	*.052 (.018 to .086)*	*.064 (.027 to .101)*	*4*	*17*

Participants indicated in italic (Group 2, Participants 8, 9, and 10) were hypothesized but not observed to demonstrate H1N1-antigen stimulated proliferation of HIV-1-infected CD4+ cells, whereas participants underlined (Group 1, Participants 1–7) were not expected to demonstrate H1N1-antigen stimulated proliferation of HIV-1-infected CD4+ cells

*ND* not detected, *NA* adequate sample not available

^a^Indicate participants that initiated ART prior to the 2009 H1N1 pandemic

The study immunization with H1N1 vaccine did not induce significant changes in HIV-1 DNA or RNA concentrations in Group 1 or Group 2 (Table 1). Across the ten women HIV-1 DNA concentrations were similar before and after vaccination (median pre- vs. post-vaccination: 96 vs. 41 copies/million PBMC, p = .37). There was also no change in the percentage of women with detectable low-level HIV RNA (50 vs. 33%, p = .55), among the six women that had ≥4.7 mL of plasma available from before and after vaccination. The change in HIV-1 DNA concentration was not significantly different between the women predicted to have proliferation of HIV-infected cells (median difference post vaccine +4.79 copies HIV-1/million PBMC) versus those that were not (median difference post vaccine −5.73 copies HIV-1/million PBMC; p = .78).

A median of 20.5 (range 18–36) HIV-1 *env* (C2-V5) sequences were generated from each participant’s pre- and post-vaccine PBMC specimen, with a total of 437 sequences across the ten subjects. Post-vaccine, participants predicted to have H1N1 vaccine-induced proliferation of HIV-infected cells had a median of a 21% decrease in the percentage of monotypic proviral sequences versus a median of a 32% increase in the percentage of monotypic proviral sequences in those not expected to have proliferation of HIV-infected cells after H1N1 vaccination, although this difference was not significantly different (p = .21) (Table 1).

A comparison of HIV-1 DNA sequences from pre- to post-vaccination did not detect differences between the groups. The median change in *env* sequence divergence from the most recent common ancestor (MRCA) was 0 in Group 1 and .2% in Group 2 (p = .27). The median change in diversity of the *env* sequences was 0 in Group 1 and .5% in Group 2 (p = .48) (Table 1; Additional file 1).

## Discussion

This study looked for evidence that an immunologic recall response by HIV-1 infected T-cells would amplify the HIV-1 proviral reservoir. None of the parameters evaluated demonstrated evidence of antigen-specific proliferation HIV-infected cells following vaccination. Specifically, the participants who were HIV-infected but not on ART during the H1N1 influenza pandemic and had H1N1 immunity prior to H1N1 vaccination did not have an increase in HIV-1 DNA load, proportion of monotypic sequences, or HIV-1 DNA divergence, after H1N1 vaccination.

This was a small study that sought to use existing specimens from a unique cohort that received a timed exposure to a recall antigen. Despite the relatively small size of the study, and the fact that all participants were pregnant women, the results are consistent with another recent study, mostly in men, which did not show a change in HIV DNA load after vaccination [12]. The lack of detectable antigen-driven proliferation in our study suggests that the timing, quantity, or route of antigen exposure by the vaccine was insufficient to alter the HIV proviral population enough to be detected by our methods [12]. We evaluated specimens 21 days after vaccination because that was the time-point at which specimens were collected in the parent study, although peak CD4 T-cell responses may be closer to 10–14 days after vaccination. Despite generating 437 bi-directional HIV *env* sequences from specimens diluted to single viral templates (a median of 20 individual HIV *env* sequences per participant before and after vaccination), the power needed to detect antigen-driven proliferation of HIV-infected cells is unknown. A recent study showed increases in HIV transcription after vaccination [12], which suggests that more intensive sampling, perhaps earlier after the exposure to antigen, and/or increased assay sensitivity, may be needed to detect antigen-driven proliferation of HIV-infected cells. Another possibility is that antigen-driven proliferation of HIV-1-infected cells may not have been detected if HAI H1N1 titers were not sensitive or specific for previous H1N1 exposure. Because there were a limited number of cells remaining available from these timepoints and H1N1 CD4 epitopes overlapped with those of other recently circulating influenza strains, we used HAI antibody titers as a proxy for CD4+ T-cell responses. It is possible that CD4 responses to previous influenza infections may not correlate closely enough with measured antibody responses to hemagglutinin. Alternatively, our underlying hypothesis may by wrong, and identical HIV-1 proviruses [2, 4] may arise due to different mechanisms. For example, the proportion of monotypic sequences may increase over time on ART from homeostatic proliferation or dysregulated proliferation of cells with HIV integrated into genes that regulate cell growth.

In summary, a recall antigen, H1N1 influenza vaccine, did not stimulate detectable proliferation of HIV-1 infected cells. This supports the safety of routine influenza vaccination in HIV-1-infected patients on ART. However, to definitively determine whether antigen-driven proliferation of infected cells is a mechanism of HIV persistence, more sensitive methods or alternative study designs may be needed.

## Additional file

**Additional file 1.** Additional figures.

## Abbreviations

HIV-1: human immunodeficiency virus type-1
ART: antiretroviral treatment
PBMC: peripheral blood mononuclear cell
H1N1: influenza A virus subtype H1N1
MRCA: most recent common ancestor
HAI: hemagglutination inhibition assay
IMPAACT: international maternal pediatric adolescent aids clinical trials network
