# Supplementary figures and images for: H1N1 influenza vaccination in HIV-infected women on effective antiretroviral treatment did not induce measurable antigen-driven proliferation of the HIV-1 proviral reservoir
Source: AIDS Res Ther. 2017 Feb 13;14:7. doi: 10.1186/s12981-017-0135-1 (PMC5307755; doi:10.1186/s12981-017-0135-1)

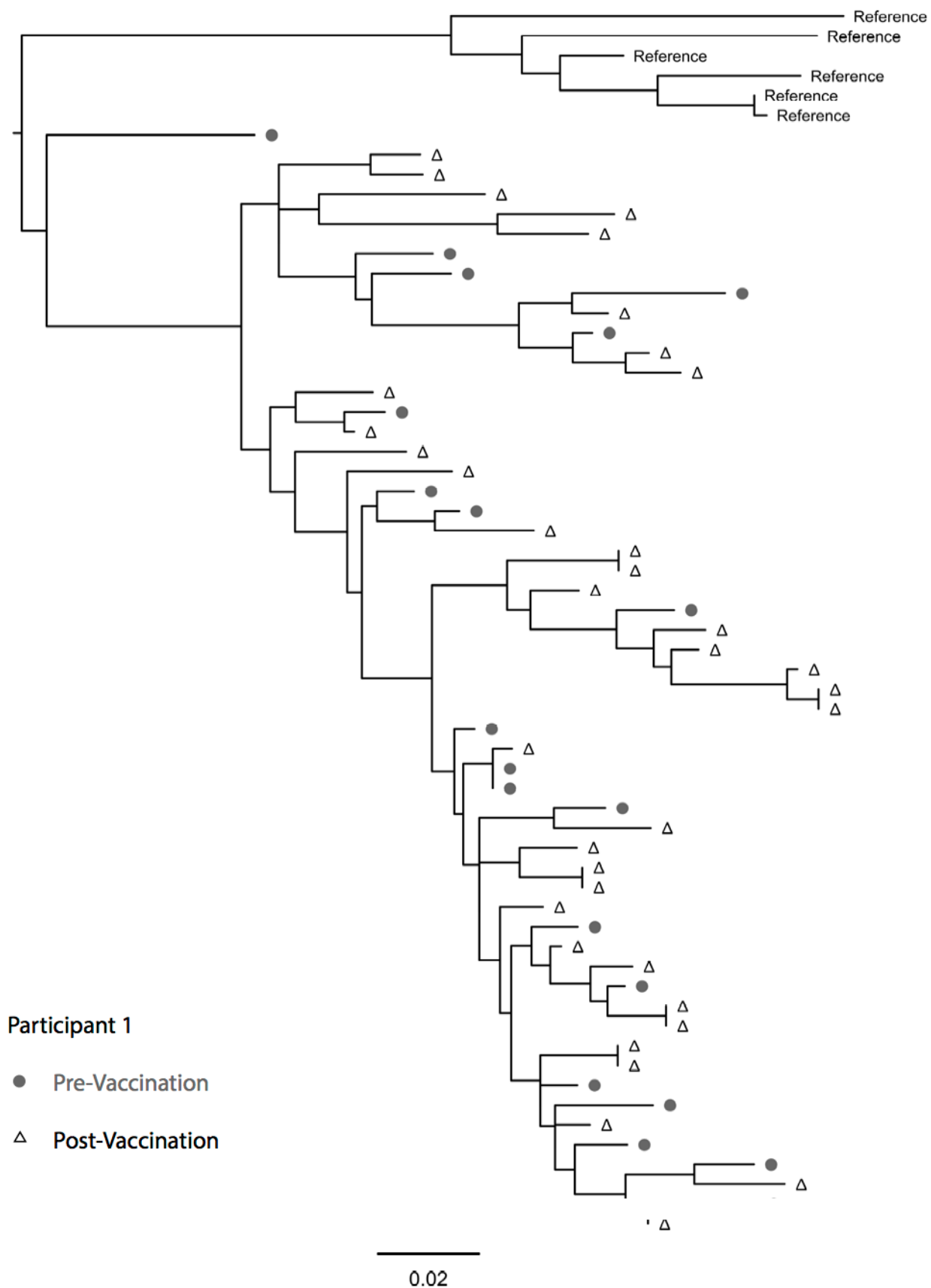

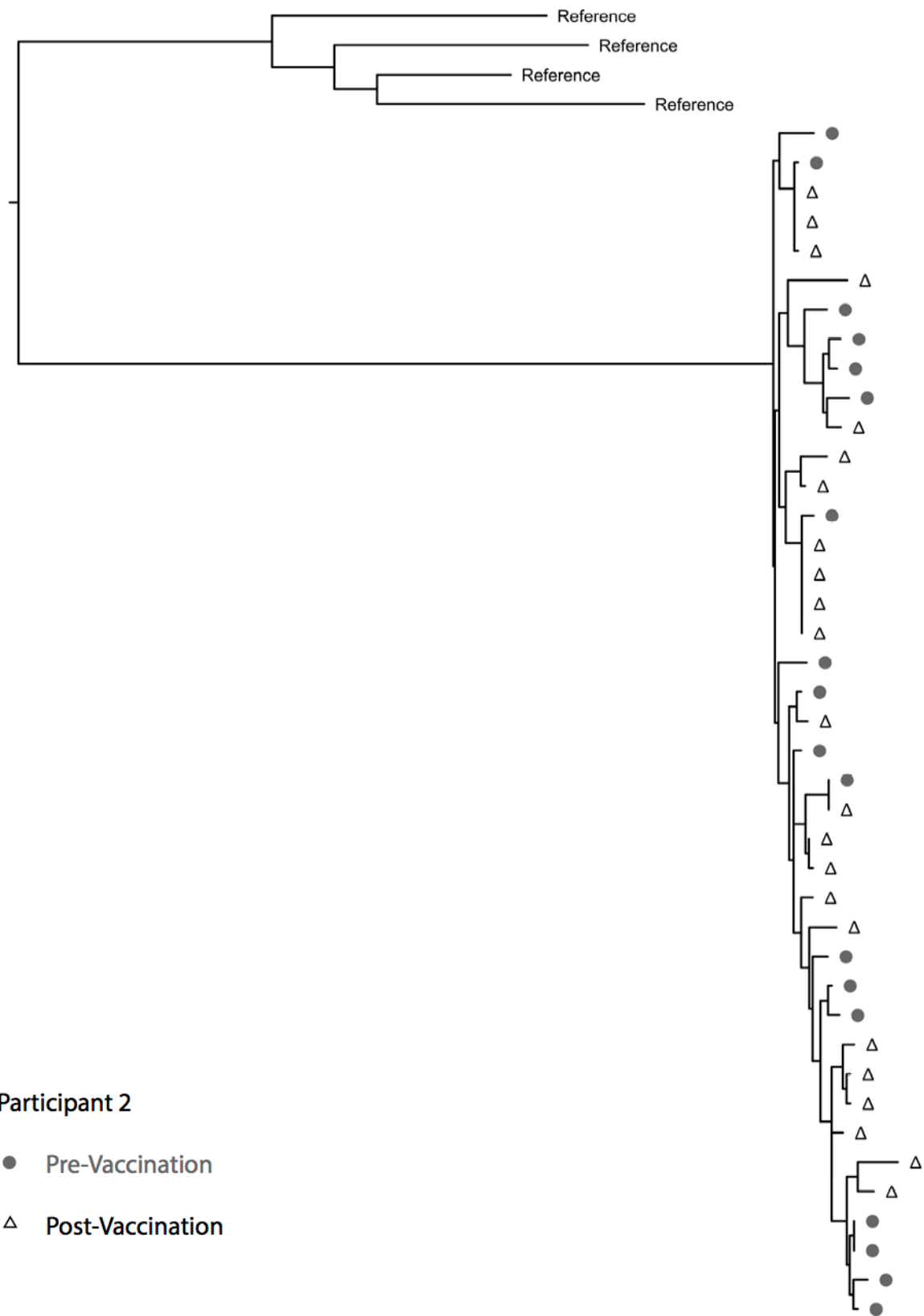

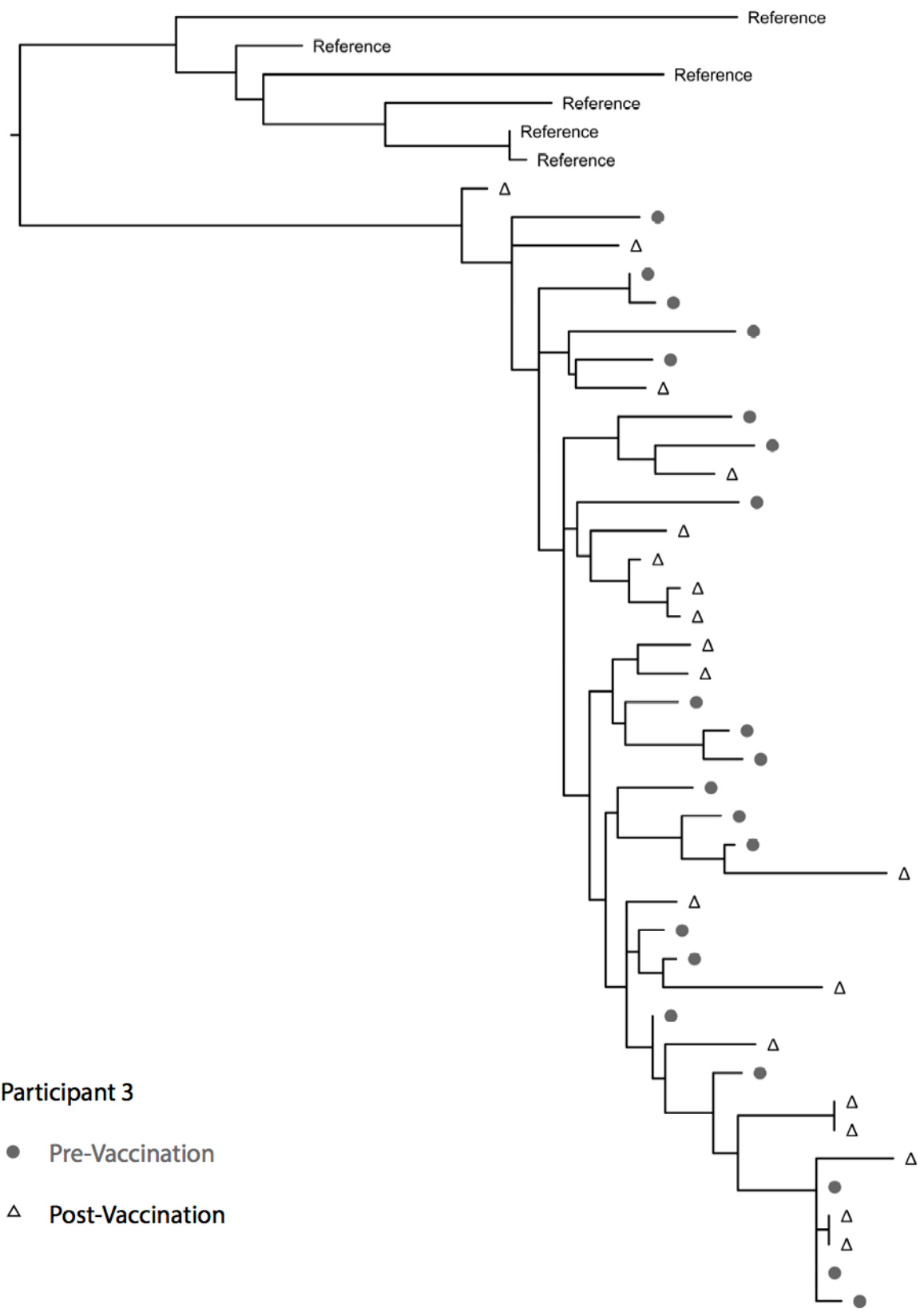

Participant 3

● Pre-Vaccination

△ Post-Vaccination

0.02







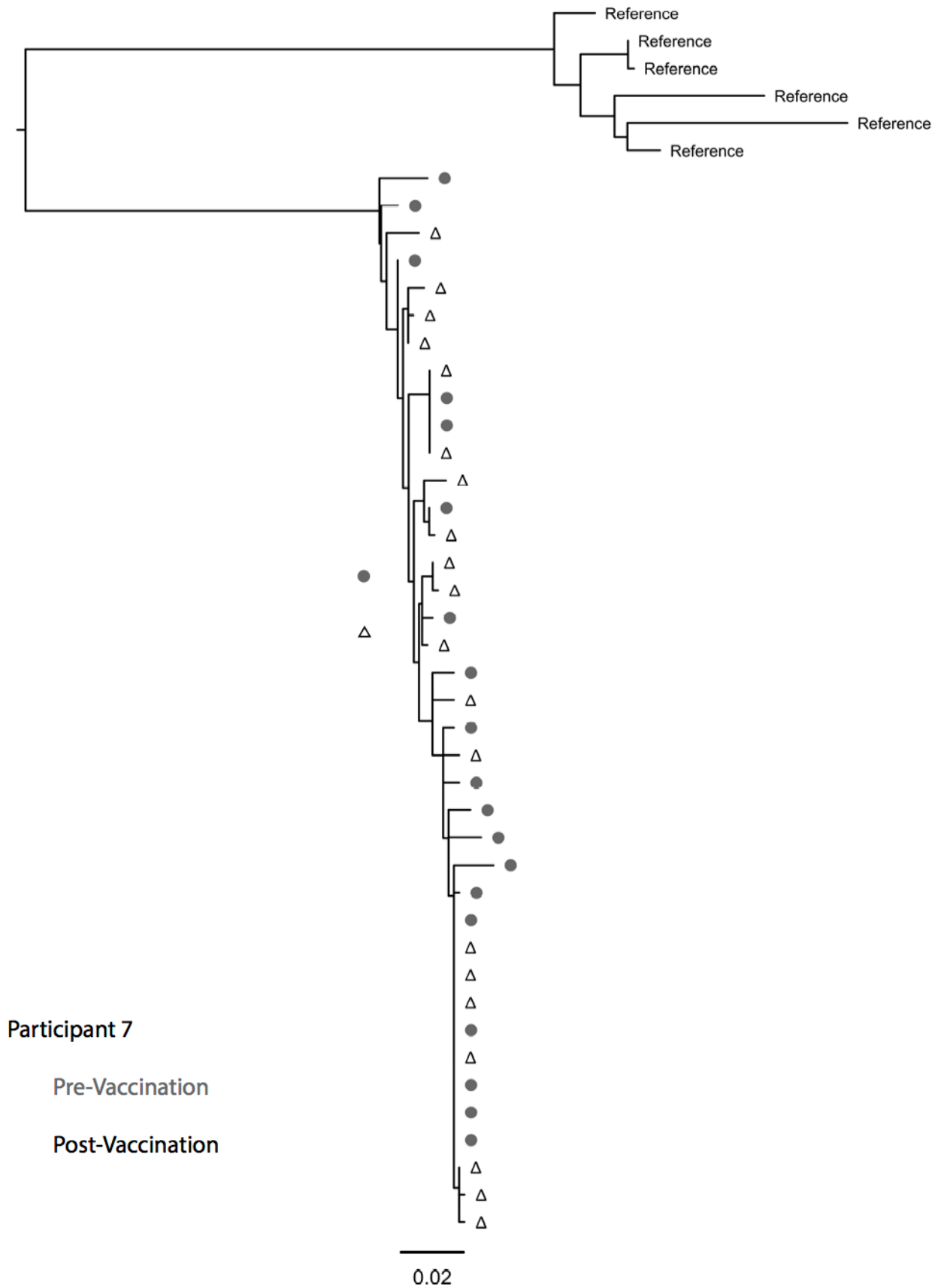



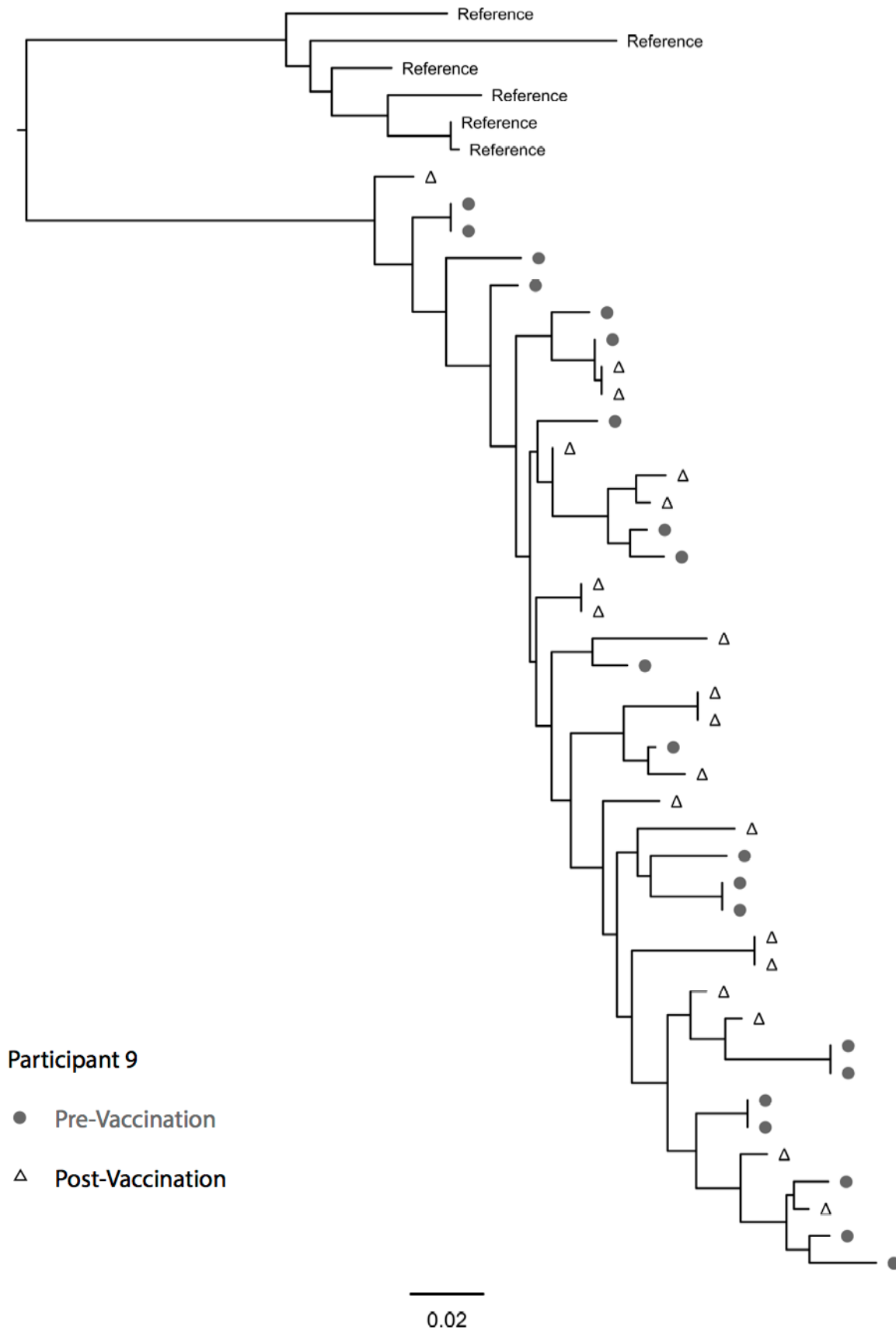

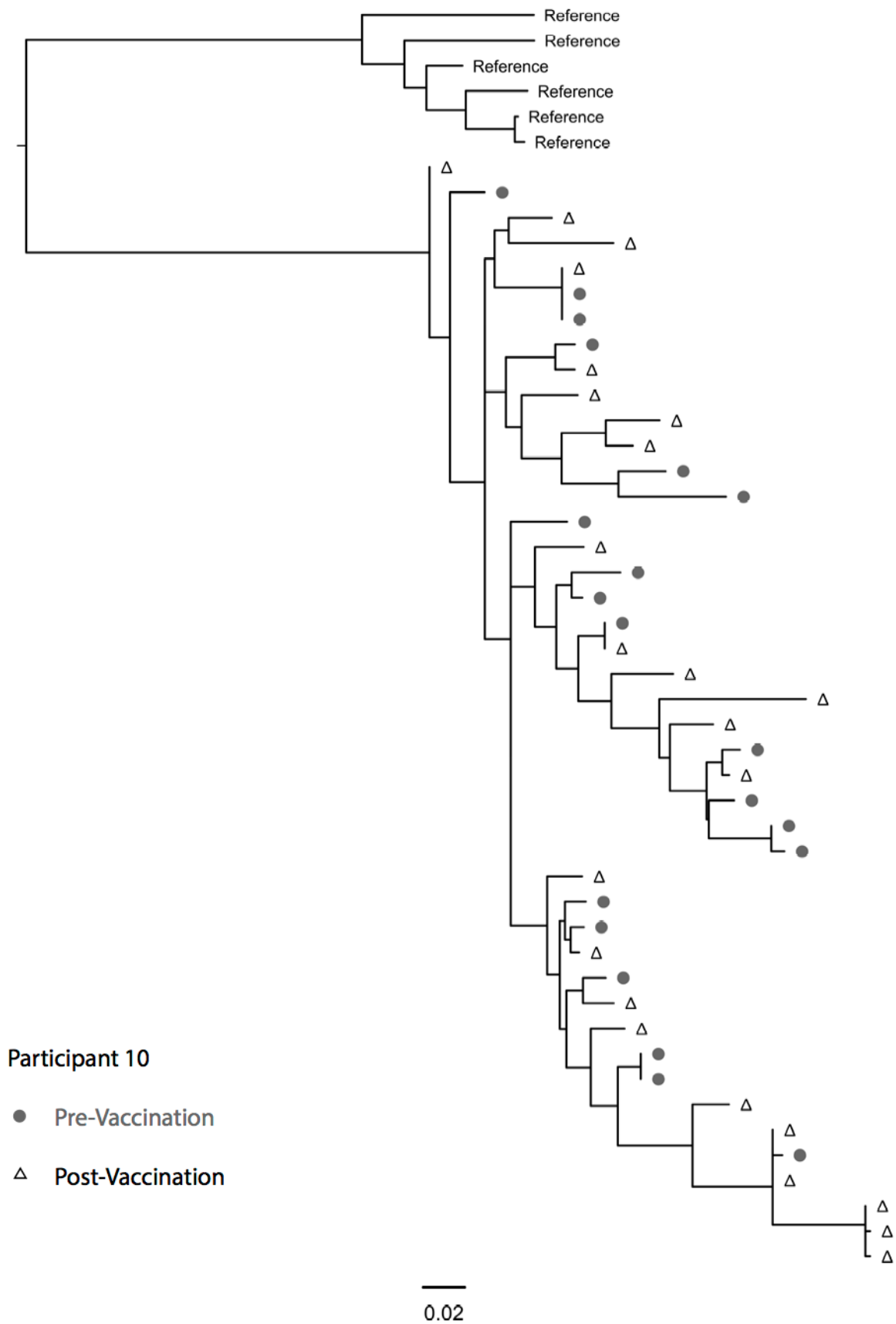

Supplement: Supplementary file 1 — Additional file 1. Additional figures. [file 12981_2017_135_MOESM1_ESM.pdf]
